# Trombose de Prótese após Valve-in-Valve Mitral: Incidência e Conduta

**DOI:** 10.36660/abc.20260330

**Published:** 2026-09-21

**Authors:** Marcos Merula de Almeida, Vitor Emer Egypto Rosa, José Honório de Almeida Palma da Fonseca, Henrique Barbosa Ribeiro, Roney Orismar Sampaio, Flávio Tarasoutchi

**Affiliations:** 1 Hospital das Clínicas Faculdade de Medicina Universidade de São Paulo São Paulo SP Brasil Instituto do Coração do Hospital das Clínicas da Faculdade de Medicina da Universidade de São Paulo, São Paulo, SP – Brasil

**Keywords:** Valva Mitral, Trombose, Anticoagulantes, Implante de Prótese de Valva Cardíaca

## Introdução

A abordagem cirúrgica constitui o tratamento convencional da disfunção de bioprótese mitral, porém as reoperações aumentam significativamente a morbimortalidade.^1^ A técnica de valve-in-valve mitral (MViV) emergiu como uma alternativa segura e eficaz para pacientes com risco cirúrgico elevado ou fragilidade biológica. Registros internacionais demonstraram elevada taxa de sucesso técnico, melhora significativa da classe funcional e bons resultados clínicos após MViV.^2-4^

Apesar dos resultados animadores, a trombose de prótese valvar (TPV) após o MViV é uma complicação com incidência global pouco conhecida e de manejo desafiador devido à escassez de dados robustos na literatura.^2,3^ Diferentemente das próteses cirúrgicas mecânicas (onde a fisiopatologia é predominantemente dependente de fluxo e hipercoagulabilidade sistêmica), a TPV em endopróteses transcateter combina fatores hemodinâmicos locais (como a perturbação de fluxo no neo-seio), subexpansão ou assimetria da estrutura, e ausência de endotelização precoce.^1^ O objetivo deste estudo é avaliar a incidência de TPV em uma coorte consecutiva pós-MViV e descrever a evolução clínica com a estratégia de anticoagulação intensificada.

## Métodos

Este estudo de registro observacional e retrospectivo incluiu inicialmente 105 pacientes submetidos ao procedimento de MViV durante 2 anos. O protocolo de pesquisa foi aprovado pelo Comitê de Ética em Pesquisa (parecer nº 4530/17/030). No seguimento de um ano, 3 pacientes foram perdidos por mudança de domicílio ou perda de contato telefônico, resultando em uma amostra final de 102 pacientes com acompanhamento clínico e ecocardiográfico completo antes da alta hospitalar, aos 30 dias e a 1 ano pós-intervenção.

A indicação de MViV foi limitada a pacientes com alto risco cirúrgico, conforme definido pelo EuroSCORE II e pelo escore de risco da Society of Thoracic Surgeons (STS), ou considerados frágeis pelo Heart Team.

A anticoagulação era realizada com varfarina, tendo como alvo terapêutico de razão normalizada internacional (INR, do inglês *international normalized ratio*) entre 2 e 3.

O diagnóstico de TPV foi baseado em deterioração clínica associada ao aumento > 50% do gradiente transprotético, restrição da mobilidade dos folhetos e presença de imagem compatível com trombo ao ecocardiograma transesofágico (ETE).

## Resultados

A incidência de TPV na coorte foi de 2,9% (3 de 102 pacientes). A Tabela 1 detalha as características demográficas e ecocardiográficas basais da população geral em relação ao subgrupo que desenvolveu trombose. Todos os três casos ocorreram em pacientes do sexo feminino e manifestaram-se clinicamente por piora súbita da dispneia (classe funcional NYHA III ou IV) correlacionada a um aumento > 50% dos gradientes transprotéticos ao ecocardiograma transtorácico (ETT) com Doppler. A população estudada apresentou idade média de 61,8 anos, com predomínio do sexo feminino (68,6%). Fibrilação atrial esteve presente em 83,3% dos pacientes. Os escores cirúrgicos evidenciaram população de alta complexidade clínica, com EuroSCORE II médio de 7,95% e STS médio de 8,09%. Os eventos ocorreram dentro dos primeiros 60 dias após o procedimento e sem casos adicionais até o primeiro ano de seguimento.

**Tabela 1:** – Características clínicas e ecocardiográficas basais da população do estudo e dos casos com TPV

	Coorte geral (n = 102)	Paciente 1	Paciente 2	Paciente 3
**Características clínicas**				
Idade (anos)	61,82	68	73	68
Sexo feminino, n (%)	70 (68,6%)	Sim (100%)	Sim (100%)	Sim (100%)
FA crônica ou paroxística, n (%)	85 (83,3%)	Não	Sim (100%)	Sim (100%)
Diabetes mellitus, n (%)	18 (17,6%)	Não	Não	Não
Hipertensão arterial sistêmica, n (%)	51 (50,0%)	Sim (100%)	Sim (100%)	Não
Tabagismo ativo, n (%)	21 (20,5%)	Sim (100%)	Não	Não
DPOC, n (%)	9 (8,8%)	Sim (100%)	Não	Não
AVCi prévio, n (%)	22 (21,5%)	Não	Não	Sim (100%)
DAOP, n (%)	2 (1,9%)	Não	Não	Não
TFGe < 60 mL/min/1,73 m^2^, n (%)	38 (37,2%)	Sim (100%)	Sim (100%)	Não
EuroSCORE II (%)	7,95	6,68	7,84	5,03
Escore STS (%)	8,09	10,6	5,38	4,56
**Achados ecocardiográficos pré-MViV**				
DSVE (mm)	34,37	42	29	42
DDVE (mm)	50,76	56	44	65
DAE (mm)	56,83	45	54	54
PSAP (mmHg)	61,82	54	54	51
FEVE (%)	59,39	40	65	45
Gmed AE-VE (mmHg)				
No diagnóstico da TPV / pós-tratamento	--	22 / 9	17 / 6	16 / 7

AVCi: acidente vascular cerebral isquêmico; DAE: diâmetro do átrio esquerdo; DAOP: doença arterial obstrutiva periférica; DDVE: diâmetro diastólico do ventrículo esquerdo; DPOC: doença pulmonar obstrutiva crônica; DSVE: diâmetro sistólico do ventrículo esquerdo; FA: fibrilação atrial; FEVE: fração de ejeção do ventrículo esquerdo; Gmed AE-VE: gradiente diastólico médio entre o átrio esquerdo e o ventrículo esquerdo; MViV: valve-in-valve mitral; PSAP: pressão sistólica da artéria pulmonar; STS: Society of Thoracic Surgeons; TFGe: taxa de filtração glomerular estimada; TPV: trombose de prótese valvar.

### Caso 1

Paciente de 68 anos, hipertensa, ritmo sinusal, com disfunção de bioprótese cirúrgica de pericárdio bovino nº 27 implantada há 6 anos. Apresentou TPV aos 28 dias pós-MViV com INR de 2,2 (STS 10,6). O ETE revelou redução da mobilidade dos folhetos e imagem sugestiva de trombo (9 × 7 mm), com gradiente diastólico médio entre o átrio esquerdo e o ventrículo esquerdo (Gmed AE-VE) de 22 mmHg.

### Caso 2

Paciente de 73 anos, com fibrilação atrial e nefropatia dialítica, portadora de bioprótese porcina nº 25 (segunda troca cirúrgica por febre reumática) com disfunção há 4 anos. Submetida a MViV via transapical (STS 5,38). Evoluiu com TPV aos 60 dias pós-procedimento com INR de 2,1; o ETE evidenciou imagem hiperecogênica aderida ao folheto lateral da endoprótese (10 × 17 mm) e Gmed AE-VE de 17 mmHg.

### Caso 3

Paciente de 68 anos, com fibrilação atrial paroxística e histórico de troca valvar mitral biológica de pericárdio bovino nº 25 há 16 anos. Realizou MViV via transapical (STS 4,56) e manifestou TPV aos 34 dias, com imagem móvel intracapsular, restrição funcional de folheto e Gmed AE-VE de 16 mmHg. INR era de 2,0.

Os casos foram conduzidos com intensificação da anticoagulação oral (INR 2,5–3,5), havendo melhora da mobilidade dos folhetos e diminuição dos gradientes (Figura 1).

**Figura 1:**
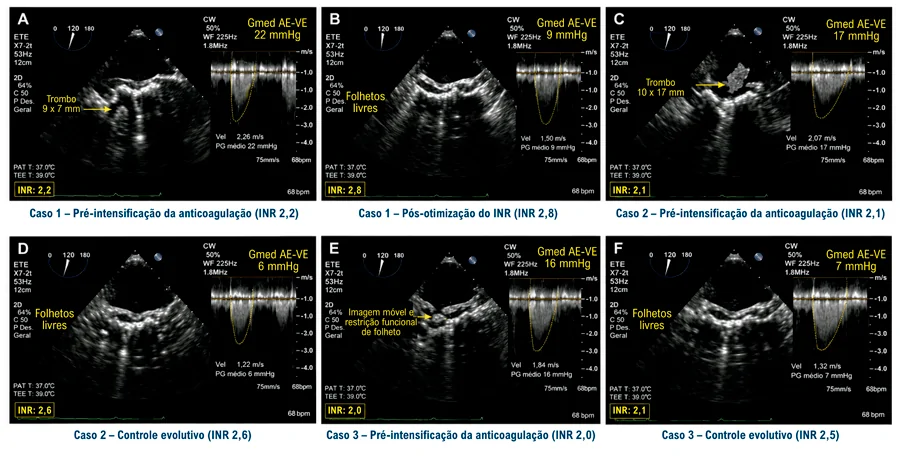
– Achados ecocardiográficos pré e pós-intensificação da anticoagulação oral. A) ETE do Caso 1 demonstrando trombo de 9 × 7 mm e gradiente médio de 22 mmHg antes da intensificação da anticoagulação. B) Após otimização do INR, houve redução do gradiente para 9 mmHg. C) ETE do Caso 2 demonstrando imagem hiperecogênica móvel de 10 × 17 mm e gradiente médio de 17 mmHg. D) Controle: normalização da mobilidade dos folhetos e gradiente de 6 mmHg. E) ETE do Caso 3 com imagem móvel e gradiente médio de 16 mmHg. F) Controle: gradiente de 7 mmHg.

## Discussão

A TPV após MViV apresenta importância clínica (incidência de 2,9% neste estudo). A literatura aponta que o uso de regimes de anticoagulação oral com antagonistas da vitamina K reduz significativamente a incidência de trombose subclínica e clínica quando comparado à terapia antiplaquetária isolada (1,6% vs. 6,6%; p = 0,019),^4,5^ conforme demonstrado em subanálises do registro SURVIV e em grandes estudos de intervenção.^1,2^

Os achados reforçam que o período inicial pós-procedimento representa fase de maior vulnerabilidade trombótica. Todos os episódios ocorreram nos primeiros 2 meses após o implante, corroborando a hipótese de endotelização incompleta e maior turbulência hemodinâmica nesse período.^4-6^

O acompanhamento clínico e ecocardiográfico seriado permite a detecção precoce da TPV. A mudança no quadro clínico deve motivar a realização de novo ETT, como ocorreu nos casos relatados.^1^

O tratamento da TPV permanece controverso. Embora a trombólise ou a reoperação cirúrgica sejam descritas em casos de instabilidade hemodinâmica, essas terapias carregam altas taxas de complicações hemorrágicas e morbimortalidade.^5,6^ Em contrapartida, a intensificação da anticoagulação oral demonstrou-se eficaz e segura, mimetizando os resultados de estudos em que se buscou o ajuste do INR (2,5–3,5). Anticoagulantes orais de ação direta ainda carecem de validação em larga escala.^5^

## Conclusão

A TPV após MViV é um evento que pode ocorrer no pós-operatório, com impacto clínico significativo. Deve ser investigado em pacientes que apresentem mudança do quadro clínico ou aumento do gradiente transprotético valvar. O incremento do nível de anticoagulação (INR 2,5–3,5) pode ser uma estratégia eficaz na resolução da trombose.
