# Supplementary material for: Trombose de Prótese após Valve-in-Valve Mitral: Incidência e Conduta
Source: Arq Bras Cardiol. 2026 Sep 21;123(9):e20260330. [Article in Portuguese] doi: 10.36660/abc.20260330 (PMC13671730; doi:10.36660/abc.20260330)
Supplement: Translation

# Translation: Prosthetic Valve Thrombosis after Mitral Valve-in-Valve: Incidence and Management

**DOI:** 10.36660/abc.20260330i

**Authors:** Marcos Merula de Almeida, Vitor Emer Egypto Rosa, José Honório de Almeida Palma da Fonseca, Henrique Barbosa Ribeiro, Roney Orismar Sampaio, Flávio Tarasoutchi

**Affiliations:** 1 Brazil Instituto do Coração do Hospital das Clínicas da Faculdade de Medicina da Universidade de São Paulo, São Paulo, SP – Brazil

**Keywords:** Mitral Valve, Thrombosis, Anticoagulants, Heart Valve Prosthesis Implantation

## Introduction

Surgical intervention is the conventional treatment for mitral bioprosthesis dysfunction; however, reoperations are associated with significantly increased morbidity and mortality.^1^ The mitral valve-in-valve (MViV) technique has emerged as a safe and effective alternative for patients at high surgical risk or those with frailty. International registries have demonstrated high technical success rates, significant improvement in functional class, and favorable clinical outcomes following MViV.^2-4^

Despite promising results, prosthetic valve thrombosis (PVT) after MViV is a complication whose overall incidence remains unclear, and its management poses challenges due to a lack of robust data in the literature.^2, 3^ Unlike surgical mechanical prostheses (where pathophysiology is predominantly driven by flow dynamics and systemic hypercoagulability), PVT in transcatheter prostheses involves a combination of local hemodynamic factors (such as flow disturbance within the neo-sinus), structural underexpansion or asymmetry, and a lack of early endothelialization.^1^ The objective of this study was to evaluate the incidence of PVT in a consecutive cohort of patients undergoing MViV and to describe the clinical course with an intensified anticoagulation strategy.

## Methods

This retrospective observational registry study initially included 105 patients who underwent the MViV procedure over a 2-year period. The research protocol received approval from the institutional research ethics committee (opinion number 4530/17/030). At the 1-year follow-up, 3 patients were lost due to relocation or inability to establish telephone contact, resulting in a final sample of 102 patients with complete clinical and echocardiographic follow-up prior to hospital discharge, as well as at 30 days and 1 year post-intervention.

The indication for MViV was limited to patients at high surgical risk, as defined by EuroSCORE II and the Society of Thoracic Surgeons (STS) risk score, or those considered frail by the Heart Team.

Anticoagulation was managed with warfarin, targeting an international normalized ratio (INR) between 2 and 3.

Diagnosis of PVT was based on clinical deterioration associated with a > 50% increase in the prosthetic valve gradient, restricted leaflet mobility, and imaging findings consistent with a thrombus on transesophageal echocardiography (TEE).

## Results

The incidence of PVT in the cohort was 2.9% (3 of 102 patients). Table 1 details the baseline demographic and echocardiographic characteristics of the overall population and the subgroup that developed thrombosis. All three cases occurred in women and manifested clinically as a sudden worsening of dyspnea (New York Heart Association functional class III or IV), correlated with a > 50% increase in the prosthetic valve gradient measured by transthoracic echocardiography (TTE) with Doppler. The study population had a mean age of 61.8 years, and women represented the majority of the population (68.6%). Atrial fibrillation was present in 83.3% of patients. Surgical risk scores reflected a population with high clinical complexity, with a mean EuroSCORE II of 7.95% and a mean STS score of 8.09%. The events occurred within the first 60 days after the procedure, with no additional cases observed up to the first year of follow-up.

**Table 1:** – Baseline clinical and echocardiographic characteristics of the study population and cases with PVT

	Overall cohort (n = 102)	Patient 1	Patient 2	Patient 3
**Clinical characteristics**				
Age (years)	61.82	68	73	68
Female sex, n (%)	70 (68.6%)	Yes (100%)	Yes (100%)	Yes (100%)
Chronic or paroxysmal AF, n (%)	85 (83.3%)	No	Yes (100%)	Yes (100%)
Diabetes mellitus, n (%)	18 (17.6%)	No	No	No
Arterial hypertension, n (%)	51 (50.0%)	Yes (100%)	Yes (100%)	No
Active smoker, n (%)	21 (20.5%)	Yes (100%)	No	No
COPD, n (%)	9 (8.8%)	Yes (100%)	No	No
Prior ischemic stroke, n (%)	22 (21.5%)	No	No	Yes (100%)
PAD, n (%)	2 (1.9%)	No	No	No
eGFR < 60 mL/min/1.73 m^2^, n (%)	38 (37.2%)	Yes (100%)	Yes (100%)	No
EuroSCORE II (%)	7.95	6.68	7.84	5.03
STS score (%)	8.09	10.6	5.38	4.56
**Echocardiographic findings before MViV**				
LVESD (mm)	34.37	42	29	42
LVEDD (mm)	50.76	56	44	65
LAD (mm)	56.83	45	54	54
PASP (mmHg)	61.82	54	54	51
LVEF (%)	59.39	40	65	45
Mean transmitral diastolic gradient (mmHg)				
At PVT diagnosis / after treatment	--	22 / 9	17 / 6	16 / 7

AF: atrial fibrillation; COPD: chronic obstructive pulmonary disease; eGFR: estimated glomerular filtration rate; LAD: left atrial diameter; LVEDD: left ventricular end-diastolic diameter; LVESD: left ventricular end-systolic diameter; LVEF: left ventricular ejection fraction; MViV: mitral valve-in-valve; PAD: peripheral artery disease; PASP: pulmonary artery systolic pressure; PVT: prosthetic valve thrombosis; STS: Society of Thoracic Surgeons.

### Case 1

A 68-year-old female patient with hypertension and sinus rhythm presented with dysfunction of a size 27 bovine pericardial surgical bioprosthesis implanted 6 years prior. She developed PVT 28 days after the MViV procedure, with an INR of 2.2 (STS score 10.6). TEE showed reduced leaflet mobility and an image suggestive of a thrombus (9 × 7 mm), with a mean transmitral diastolic gradient of 22 mmHg.

### Case 2

A 73-year-old female patient with atrial fibrillation and dialysis-dependent kidney disease had a size 25 porcine bioprosthesis (second surgical replacement due to rheumatic fever) that had been dysfunctional for 4 years. She underwent transapical MViV (STS score 5.38) and developed PVT 60 days after the procedure, with an INR of 2.1. TEE revealed a hyperechoic image adherent to the lateral leaflet of the endoprosthesis (10 × 17 mm) and a mean transmitral diastolic gradient of 17 mmHg.

### Case 3

A 68-year-old female patient with paroxysmal atrial fibrillation had undergone mitral valve replacement with a size 25 bovine pericardial bioprosthesis 16 years prior. She underwent transapical MViV (STS 4.56) and developed PVT 34 days after the procedure, with an intracapsular mobile mass, restricted leaflet motion, a mean transmitral diastolic gradient of 16 mmHg, and an INR of 2.0.

The cases were managed by intensifying oral anticoagulation (INR 2.5–3.5), resulting in improved leaflet mobility and reduced gradients (Figure 1).

**Figure 1:** – Echocardiographic findings before and after intensification of oral anticoagulation. A) TEE of Case 1 showing a 9 × 7 mm thrombus and a mean gradient of 22 mmHg prior to anticoagulation intensification. B) After INR optimization, the gradient decreased to 9 mmHg. C) TEE of Case 2 showing a mobile hyperechoic mass measuring 10 × 17 mm and a mean gradient of 17 mmHg. D) Follow-up: normalization of leaflet mobility and a gradient of 6 mmHg. E) TEE of Case 3 showing a mobile mass and a mean gradient of 16 mmHg. F) Follow-up: gradient of 7 mmHg. INR: international normalized ratio; MG: mean transmitral diastolic gradient; TEE: transesophageal echocardiography.

## Discussion

PVT following MViV is clinically significant (incidence of 2.9% in this study). The literature indicates that oral anticoagulation regimens using vitamin K antagonists significantly reduce the incidence of subclinical and clinical thrombosis compared to antiplatelet therapy alone (1.6% vs. 6.6%; p = 0.019),^4,5^ as demonstrated in subanalyses of the SURVIV registry and in large interventional studies.^1,2^

These findings reinforce that the early postprocedural period represents a phase of heightened thrombotic vulnerability. All episodes occurred within the first 2 months after implantation, supporting the hypothesis of incomplete endothelialization and increased hemodynamic turbulence during this period.^4-6^

Serial clinical and echocardiographic follow-up allows for the early detection of PVT. Changes in clinical status should prompt a repeat TTE, as in the reported cases.^1^

The treatment of PVT remains controversial. Although thrombolysis and surgical reintervention have been described for cases of hemodynamic instability, these therapies carry high rates of bleeding complications, morbidity, and mortality.^5,6^ Conversely, intensified oral anticoagulation has proven effective and safe, mirroring results from studies targeting an adjusted INR between 2.5 and 3.5. Direct oral anticoagulants still lack large-scale validation.^5^

## Conclusion

PVT following MViV is an event that may occur during the postoperative period, with significant clinical impact. It should be investigated in patients presenting with changes in clinical status or an increase in the prosthetic valve gradient. Increasing the level of anticoagulation (INR 2.5–3.5) may be an effective strategy for resolving the thrombosis.
